# Satisfaction of Nigerian patients with health services: a protocol for a systematic review

**DOI:** 10.1186/s13643-019-1160-z

**Published:** 2019-11-01

**Authors:** Maureen O. Akunne, Mathew J. Okonta, Chinwe V. Ukwe, Thomas L. Heise, Obinna I. Ekwunife

**Affiliations:** 1 Department of Clinical Pharmacy and Pharmacy Management, University of Nigeria, Nsukka, Enugu State Nigeria; 2 Research Group for Evidence-Based Public Health, Institute for Public Health and Nursing Research, Health Sciences, University of Bremen, Bremen, Germany; 3 Leibniz Institute for Prevention Research and Epidemiology – BIPS, Bremen, Germany; 4 Department of Clinical Pharmacy and Pharmacy Management, Nnamdi Azikiwe University, Awka, Anambra State Nigeria

**Keywords:** Patient satisfaction, Health services, Nigeria, Systematic review

## Abstract

**Background:**

Patient-based assessment of health services is becoming popular in measuring the standard of care. Both quantitative and qualitative methods are available. Patient satisfaction surveys are commonly used to record the experiences of patients in hospitals, whereas qualitative designs (e.g., interviews and focus group discussions) are used less frequently. To date, there has been no systematic review published devoted to patient satisfaction with health services in Nigeria. We aim to (1) systematically analyze relevant quantitative studies to pinpoint excellent procedures in measuring patient satisfaction with health services, (2) to investigate if a reference method (gold standard method) exists, and (3) to identify relevant topics which are recognized by patients as important for the delivery of a high-quality health service in Nigeria.

**Methods:**

Searches of eight electronic journal databases, including MEDLINE, EMBASE, CINAHL, PsycINFO, AJOL, CDSR, DARE, and HTA will be conducted to identify studies assessing patient satisfaction with health services in Nigeria. The searches will be supported by manual searches in reference lists of relevant primary studies and systematic reviews. The review will be limited to studies published since 2007. After a stepwise screening process by two reviewers, data from included studies will be extracted and reviewed. The COSMIN RoB checklist will be used to critically appraise included studies. We will carry out an extensive data synthesis to answer the review questions.

**Discussion:**

The intended systematic review will provide information on how the satisfaction of patients with health services has earlier been described and assessed in Nigerian studies. It will establish if a gold standard method exists and synthesize information on topics which might be of special interest to patients. Review findings will enrich the debate on patient-centered care and overall performance of health quality standards in Nigeria.

**Systematic review registration:**

PROSPERO CRD42018108140

## Background

### The Nigerian patients and health services

Low- and middle-income countries (LMICs) such as Nigeria commonly face the dual health burden caused by infectious (communicable) diseases (e.g., malaria, HIV/AIDS, hepatitis, tuberculosis, influenza) and non-infectious (non-communicable) diseases (e.g., diabetes mellitus, cancer, cardiovascular diseases, chronic respiratory diseases) [1]. The nation’s healthcare system has also notable challenges which include underinvestment in health systems, poor healthcare infrastructure, inadequate funding and policy frameworks, and insufficient implementations of Public-Private-Partnerships (PPPs). These, among other challenges, may contribute to poor satisfaction with health services experienced by Nigerian patients [2, 3].

Many Nigerian patients, those who can afford it, now receive medical treatment abroad (medical tourism), especially for general surgeries as well as disease management in the fields of cardiology, neurology, and overall cancer management. In 2012, Nigerians spent an estimated 260 million USD in India for medical treatment and related costs alone. A large proportion of travels to India (about 40%) can be explained by medical reasons [4]. Another report also shows that Nigerians spend 1 billion USD every year to obtain emergency medical treatments abroad [5]. This includes medical tourism to countries and geographic regions such as South Africa, Zimbabwe, and countries in the Middle East (e.g., Egypt, Israel, Turkey) which are supposed to offer better health care service compared to Nigeria. It is not as if the country does not produce the required clinical expertise, but lack of favorable circumstances and enabling environments in the nation form the basis for the persistent brain drain, not to mention abnormally low remunerations. The National Health Service (NHS) in the UK, for example, has over and above 4000 physicians employed from Nigeria, this number is even higher for Nigerian doctors working in the USA. In total, more than 25,000 physicians from Nigeria are working abroad, affecting healthcare delivery back home [6]. This, without doubt, negatively influences the standard of care and could invariably affect the satisfaction of patients in Nigeria, i.e., those who cannot afford overseas treatment.

Financing of healthcare is very important to achieving universal healthcare coverage. In Nigeria, healthcare financing occurs through a variety of sources such as revenue from taxes, direct payment for treatment (out-of-pocket payments (OOPs)), funding by donors, and health insurances (social and community). WHO, UNICEF, and USAID are some of the donor groups that have funded Nigeria healthcare projects. With the inauguration of the National Health Insurance Scheme (NHIS) in Nigeria in 2005, accessibility to healthcare was provided to many Nigerians. But since then, only those working in public (formal) sectors (less than 5% of the Nigerian workforce) have registered. Nigerian government intends to use Community-Based Health Insurance (CBHIS) for workers in the informal sector and rural areas. States such as Lagos, Anambra, Ogun, and Kwara are currently implementing CBHIS. For instance, a law was passed in Lagos establishing a Lagos State Health Insurance Scheme [4].

The slow pace of Nigeria’s implementation efforts to achieve universal health insurance coverage is unfortunate because countries such as Ghana, Rwanda, and Kenya—which started several years after Nigeria—have hit over 50% coverage in the meantime [7]. Direct payments in the form of OOPs are the main source, comprising about 70.3% of total health expenditures in 2009 [8]. This percentage share is seen as one of the highest in the world [8].

### Health outcome surveys

Definition of healthcare outcomes has been given as “measures of the result of what happens to patients as a consequence of their encounters with the healthcare system” [9]. In clinical research, three types of outcomes are measured: (a) clinical outcomes which are consequences of a medical treatment (e.g., laboratory values); (b) humanistic outcomes which are end results of a disease or a treatment on the patient’s ability to perform normal daily activities and quality of life, including patient-reported outcomes (PROs) (e.g., satisfaction of patients with healthcare services); and (c) economic outcomes which are direct, indirect, and intangible costs of treatment in relation with the results of therapy options.

Traditional medical models of care had concentrated on clinical outcomes only, whereas more recent medical models of care integrate clinical outcomes as well as economic and humanistic outcomes. This new approach, the Economic, Clinical and Humanistic Outcomes is termed the ECHO model of care. The ECHO model builds on the traditional model but broadens its scope by incorporating the humanistic and economic outcomes and thus provides a more comprehensive measurement of health care value [10].

Patient satisfaction, a humanistic outcome, has been studied as a crucial element when assessing health outcomes and standard of care [11]. Often, quantitative surveys such as questionnaires have been used to document patient interactions with the healthcare system, such as their satisfaction with health services in hospitals and clinics. Qualitative designs such as interviews and focus group discussions are less often used, but can gain a more comprehensive and thorough view of patients’ experiences. A study conducted in Nigeria [11] recorded high level of satisfaction of HIV patients with pharmaceutical services using a Patient Satisfaction with Pharmaceutical Service (PSPS) questionnaire [12]. However, in the study, the patients indicated low satisfaction with the item that examined the supply of penned information by pharmacists. Using a standardized structured interviewed questionnaire, Karunamoorthi et al. [13] also demonstrated that most of the respondents were satisfied with the pharmaceutical service. 82.5% of the participants in the study, however, indicated long waiting hours to finally receive their medication as a major problem that is responsible for their dissatisfaction. The research on the satisfaction of patients with health services is not only important in chronic diseases such as HIV/AIDS, but, in fact, in all areas of health service delivery. For instance, a study done in the south-west region of Nigeria on the satisfaction with primary health services also indicated waiting time and availability of drugs as the main interests of the participants [14]. It is worthwhile to note that respondents under NHIS as well as non-NHIS respondents were highly satisfied with the health services they had received from their healthcare providers at their healthcare facilities [15–19]. Contrary to the studies discussed above, Abebe et al. recorded low levels of satisfaction by the respondents with pharmaceutical services [20]. Thus, evaluation of patients’ satisfaction with health services is important and crucial to pinpoint certain areas of service and practice which need to be improved. A systematic analysis of these relevant studies can also help to identify relevant approaches in evaluating patient satisfaction and to establish different appraisal instruments based on the patients’ most important considerations.

A review focusing on the measurement of patient satisfaction in clinics specialized in the field of sexually transmitted diseases (STDs) has already been published [21]. Some of such reviews included studies which had been carried out in LMICs such as Nigeria [22, 23]. Another review examined the satisfaction measurement instruments for healthcare service users in Brazil [24]. In the latter review, the researchers concluded, based on their findings, that there was no gold standard method for assessing patient satisfaction. A review of patients’ views on the quality of primary health care in sub-Saharan Africa was published in 2015. This review included six studies from Nigeria, but did not make country-specific conclusions [25]. Even though studies from Nigeria were included in systematic reviews before, none has been conducted focusing on Nigerian patients and their satisfaction with local health services. The study therefore intends to address the following questions:

1. How has patient satisfaction with health services in Nigerian hospitals been previously described and assessed?
2. Is there any existence of a gold standard method?
3. Is it possible to identify issues/topics considered by patients as most important in delivering a high-quality service?
4. Are there any areas of dissatisfaction and if so, what are they?

## Methods

### Protocol

This protocol follows the guidelines on reporting as outlined in the Preferred Reporting Items in Systematic Reviews and Meta-analyses (PRISMA) statement [26]. The protocol is enlisted in the International Prospective Register of Systematic Reviews (PROSPERO) CRD42018108140.

### Eligibility criteria

To be included in this upcoming review, studies must meet the following criteria:

1. Studies investigating the satisfaction of patients with health services in Nigerian hospitals or clinics;
2. Studies on adult patients, 18 years and above;
3. Studies performed with quantitative instruments (questionnaire-based studies);
4. The analysis will consider original research works and reviews (consideration of the latter is to ensure that no original work will be missed);
5. Eligible studies need to be published in English;
6. Studies assessing patient satisfaction as a primary outcome (see the “Outcome types” section);
7. Studies published between 2007 and 2018.

We will, therefore, exclude qualitative studies, studies published in other languages than English, and studies published earlier than 2007. The exclusion of studies published earlier than 2007 was due to the rapid changes of the Nigerian healthcare system over the last decades and to ensure studies were conducted after the introduction of the NHIS. We wanted to avoid study results which can be solely traced back to possible adaptation issues in the transition period shortly after the introduction of the NHIS.

### Information sources

We will perform searches in the Medical Literature Analysis and Retrieval System Online (MEDLINE; via OvidSP), Excerpta Medica database (EMBASE; via OvidSP), Cumulative Index to Nursing and Allied Health Literature (CINAHL; via EBSCOhost), PsycINFO (PsycINFO; via OvidSP), and African Journals OnLine (AJOL; via AJOL) to retrieve primary studies and systematic reviews. For existing systematic reviews, we will also conduct searches in the Cochrane Database of Systematic Reviews (CDSR; via Wiley), the Database of Abstracts of Reviews of Effects (DARE; via Wiley), and the Health Technology Assessment Database (HTA; via Wiley).

### Search strategy

Our search strategy follows a highly sensitive search approach. We will use a combination of relevant text words and Medical Subject Headings (MeSH) or other hierarchical medical vocabulary systems to incorporate basic elements of our research question, this includes population and context (e.g., hospitals, clinics, health service, and patients), geographic region (i.e., Nigeria), outcomes (e.g., patient satisfaction and perception), and study design (e.g., study, trial, and surveys). If provided by the database provider, we will use search filters to exclude animal studies and published articles before 2007. A search for MEDLINE was already developed and piloted by the author team. This MEDLINE search will be adapted to match the necessities of the other included databases. The original MEDLINE search is provided in the Appendix section below.

### Study selection

Before entering the screening process, we will de-duplicate all references by using the reference software Endnote. Screening of titles and abstracts will be independently done by two of the reviewers (MA and OE) in consideration of the eligibility criteria. A third reviewer’s opinion (MO) will be requested to reach a general agreement in case of disagreement with regard to potential in- or exclusion of studies. The same elements of the screening process will be used for the full-text screening afterwards.

### Outcome types

The primary outcome will be patient satisfaction with health services. Studies in which satisfaction is measured only as a single dimension of service provision (as in assessing the satisfaction of patients with physician interaction) will be excluded.

### Data extraction

A data extraction form will be first piloted and subsequently provided to the review team. Two reviewers (MA and OE) will separately extract relevant information from the included studies. Any disagreement will be handled by discussion and final advice from a third author (MO). Data to be extracted from each study will be relevant with regard to patient-reported outcome measures (PROMs), including patient sample and results of the measurement properties (MA, OE, and MO). Thus, the following items will be considered: year, authors, sample size, study design, healthcare setting (hospital or clinic), satisfaction tool employed, dimensions of the instruments, format, and psychometric properties analyzed.

### Risk of bias

The COnsensus-based Standards for the selection of health status Measurement INstruments (COSMIN) risk of bias checklist will be utilized to assess the validity and reliability of eligible studies. The checklist contains design necessities and approved statistical methods on measurement properties. For each of these properties, a checklist was developed containing standard questions needed to evaluate the quality of a study on that particular measurement property (referred to as COSMIN box). There are ten COSMIN boxes of the risk of bias checklist for the different measurement properties. For example, box 1 is for development, box 2 for content validity, and box 3 for structural validity. More than one property may be evaluated and documented in a study, meaning that more than one COSMIN box needs to be completed. The individual studies will be rated as either very good, adequate, doubtful, or of inadequate quality [27]. The quality of each study will be determined by taking the lowest rating of any of the standard questions (i.e., “the worst score counts” principle) [28]. Details and full explanation of the COSMIN checklist and its development process can be found in Mokkink et al. [29]. The use of the COSMIN risk of bias checklist is justified for this particular review as the checklist was exclusively developed and validated for evaluating the methodological quality of measures used to assess PROMs such as satisfaction, perception, and quality of life in health research [29]. Boxes of the COSMIN risk of bias statement are summarized in Table 1.

**Table 1:** Boxes of the COSMIN risk of bias checklist [29]

Mark the measurement properties that have been evaluated in the article^a^.	
Content validity	
Box 1. PROM development Box 2. Content validity	
Internal structure	
Box 3. Structural validity Box 4. Internal consistency Box 5. Cross-cultural validity/measurement invariance	
Remaining measurement properties	
Box 6. Reliability Box 7. Measurement error Box 8. Criterion validity Box 9. Hypotheses testing for construct validity Box 10. Responsiveness	

^a^If a box needs to be completed more than once, two or more can be placed

### Data synthesis

After reviewing published systematic reviews in this research field (see the chapter “Background” section), we do not plan to do a meta-analysis as a form of data synthesis (mostly relevant to our fourth objective) as we expect studies from a wide variety of settings (e.g., general hospitals, specialized hospitals), different populations (e.g., men, women, diseased, non-diseased), different study designs (e.g., cross-sectional studies, uncontrolled before-after studies (UBAs)), and different timings of questioning the patients (e.g., hospital discharge surveys, surveys at any given time). However, a detailed narrative synthesis will be carried out to answer our four main objectives. Information will be sought only from the studies which reported results on one or more measurement properties of the COSMIN risk of bias checklist. Important features of these studies will be presented and organized in a tabular form. Included studies will be summarized under key topics that came out during thematic analysis.

## Discussion

This protocol reports detailed information of an upcoming systematic review which will include studies on the satisfaction of patients with health services in Nigerian hospitals. The aim is to evaluate how patient satisfaction with health services has been described and measured in the setting of Nigerian hospitals over the past decade, a decade with fundamental healthcare reforms in Nigeria. The research gaps discovered through this systematic review will identify areas for further research as well as the direction for future studies. Areas of dissatisfaction within the context of Nigerian health services will also be pointed out. As a result, this review could contribute to a harmonization process of high-quality methods used in conducting quantitative surveys in this context.

## Appendix

**Table 2:** Details of one electronic bibliographic database search query (MEDLINE)

MEDLINE (via OvidSP) [1932 hits] access day 20180730	
1. exp Patient centered care/ 2. exp Hospitals/ 3. exp Health Services Research/ 4. exp Quality of Health Care/ 5. clinic*.tw. 6. hospital?.tw. 7. patient?.tw. 8. outpatient?.tw. 9. inpatient?.tw. 10. (medical adj1 (treatment or treatments or service or services or centre or centres or center or centers)).tw. 11. (health adj1 (service or services or centre or centres or center or centers)).tw. 12. health?care.tw. 13. health?system?.tw. 14. or/1-13 15. Nigeria.cp. 16. Nigeria.tw. 17. nigerian?.tw. 18. Lagos.tw. 19. Kano.tw. 20. Ibadan.tw. 21. "Benin City".tw. 22. Jos.tw. 23. Ilorin.tw. 24. Kaduna.tw. 25. Abuja.tw. 26. Enugu.tw. 27. Warri.tw. 28. or/15-27 29. exp Patient Satisfaction/ 30. acceptabilit*.tw. 31. acceptance.tw. 32. attitude?.tw. 33. dissatisf*.tw. 34. judgement*.tw. 35. satisf*.tw. 36. view?.tw. 37. opinion*.tw. 38. perception?.tw. 39. perceived.tw. 40. preference?.tw. 41. preferred.tw. 42. or/29-41 43. exp Empirical Research/ 44. exp Data Collection/ 45. assessment?.tw. 46. evaluation?.tw. 47. intervention?.tw. 48. survey*.tw. 49. study.tw. 50. studies.tw. 51. trial.tw. 52. trials.tw. 53. questionnaire?.tw. 54. or/43-53 55. 14 and 28 and 42 and 5456. limit 55 to ed=”20070101-20180730” 57. (animals not (humans and animals)).sh. 58. 56 not 57	

## Abbreviations

AIDS: Acquired immune deficiency syndrome
CBHIS: Community-Based Health Insurance Scheme
COSMIN: COnsensus-based Standards for the selection of health status Measurement INstruments
ECHO: Economic, Clinical and Humanistic Outcomes
HIV: Human immunodeficiency virus
LMICS: Low- and middle-income countries
NHIS: National Health Insurance Scheme
NHS: National Health Service
OOP: Out-Of-Pocket
PPPs: Public-private-partnerships
PRISMA: Preferred Reporting Items in Systematic Reviews and Meta-analyses
PRO: Patient-reported outcomes
PROMS: Patient-reported outcome measures
PSPS: Patient Satisfaction with Pharmaceutical Service
